# Functional Relationship between Inhibitory Control, Cognitive Flexibility, Psychomotor Speed and Obesity

**DOI:** 10.3390/brainsci12081080

**Published:** 2022-08-15

**Authors:** Marco La Marra, Ciro Rosario Ilardi, Ines Villano, Mario Carosella, Maria Staiano, Alessandro Iavarone, Sergio Chieffi, Giovanni Messina, Rita Polito, Alessia Scarinci, Vincenzo Monda, Girolamo Di Maio, Antonietta Messina

**Affiliations:** 1 Department of Experimental Medicine, University of Campania “Luigi Vanvitelli”, 80138 Naples, Italy; 2 Department of Psychology, University of Campania “Luigi Vanvitelli”, 81100 Caserta, Italy; 3 Neurological Unit, CTO Hospital, AORN “Ospedali dei Colli”, 80131 Naples, Italy; 4 Department of Clinical and Experimental Medicine, University of Foggia, 71122 Foggia, Italy; 5 Department of Education Sciences, Psychology, and Communication, University of Bari, 70121 Bari, Italy; 6 Department of Movement Sciences and Wellbeing, University of Naples “Parthenope”, 80138 Naples, Italy

**Keywords:** morbid obesity, executive functions, inhibitory control, verbal fluency, psychomotor speed

## Abstract

In the last decades, it has been proposed that executive functions may be particularly vulnerable to weight-related issues. However, evidence on the matter is mixed, especially when the effects of sociodemographic variables are weighted. Thus, the current study aimed at further examining the relationship between executive functions and obesity. To this aim, we compared treatment-seeking overweight, obese, and morbidly obese patients with normal-weight control participants. We examined general executive functioning (Frontal Assessment Battery–15) and different executive subdomains (e.g., inhibitory control, verbal fluency, and psychomotor speed) in a clinical sample including 208 outpatients with different degrees of BMI (52 overweight, BMI 25–30, M age = 34.38; 76 obese, BMI 30–40, M age = 38.00; 80 morbidly obese, BMI > 40, M age = 36.20). Ninety-six normal-weight subjects served as controls. No difference on executive scores was detected when obese patients were compared with over- or normal-weight subjects. Morbidly obese patients reported lower performance on executive scores than obese, overweight, and normal-weight subjects. Between-group difference emerged also when relevant covariates were taken into account. Our results support the view that morbid obesity is associated with lower executive performance, also considering the critical role exerted by sociodemographic (i.e., sex, age, and education) variables. Our results support the view that executive functioning should be accounted into the management of the obese patient because of non-negligible clinical relevance in diagnostic, therapeutic, and prognostic terms.

## 1. Introduction

Obesity represents a strong concern for public health [1] since it is linked to decreased quality of life and worse prognosis for concomitant chronic diseases [2]. Moreover, obesity is a major risk factor for several morbidities [3,4,5,6]. Recently, scientific research has looked beyond the physical consequences of obesity and sought to explore the association between obesity and cognitive processes [7]. Indeed, it appears that obesity predicts adverse neurocognitive outcomes [8]. More specifically, obesity would increase the risk of cognitive defects, besides being a potential predictor of cognitive decline and mild/major neurocognitive disorders [9,10,11,12,13,14,15,16,17,18,19].

The first generation of studies investigating the relationship between obesity and cognition found that obese patients performed worse on tasks assessing global cognitive functioning, with particular reference to attention and memory [20,21], showing also higher longitudinal decline than their leaner peers [22,23,24]. Subsequently, the same research line has moved the attention towards executive function (EFs). Strictly related to neural activity within the prefrontal cortex (PFC), with particular reference to dorsolateral and orbitofrontal cortices, EFs represent higher cognitive processes supporting cognitive control [25,26] and goal-directed behaviours [27]. EFs embrace several domains (e.g., attention, processing speed, set-shifting, inhibitory control, working memory, concept formation, and problem solving) [28,29,30,31,32] involved in applying adaptive behaviors to efficiently face environmental demands, especially in conflicting contexts [28,30,33]. Particularly, EFs act to inhibit dominant responses or interfering stimuli, and to resist temptations [27].

The prefrontal cortex, in concert with mesolimbic structures, is engaged in the food self-regulation process related to reward mechanisms and in processing the strong hedonic component of food choice [25,34]. Interestingly, inefficiency of frontal/executive processes was found to be associated with weak food inhibition [35,36,37], increased fatty foods intake [38], poor appetite regulation [25], insufficient physical activity [39], higher emotional eating [40], delay in weight loss [35], and lower adherence to, and unsatisfactory outcomes following, dietary interventions [41,42]. These findings are supported by neuroimaging studies showing hypoperfusion within frontal territories and the adjacent portions of temporal and parietal cortices in patients with a higher body mass index (BMI) [43,44,45]. However, some investigations have reported conflicting evidence suggesting that obese subjects would get, in line with the “obesity paradox” [46,47], equal or better performance than normal-weight subjects on executive tasks [24,48,49,50].

Previous research on obesity has provided mixed evidence, especially over the inhibitory control, verbal fluency, and psychomotor speed domains [7,25,51,52,53,54]. Inhibitory control allows the voluntary suppression of prepotent/habitual responses in line with task demands. Although some studies have shown that obese subjects demonstrated lower inhibitory control than normal-weight subjects [24,35,54], other studies have found no relationship between obesity and inhibition processes [7,55,56,57]. Verbal fluency enables spontaneous retrieval of specific information within phonemic or semantic constraints [58,59]. Although some studies have found that obese subjects performed worse than non-obese ones [24,60], other investigations have shown no between-group differences [61] or even better performance in obese participants [7,48,54]. Finally, psychomotor speed underlies the ability to detect and respond to rapid changes in the environment, such as the presence of a stimulus [59]. Also in the latter case, some studies have reported lower performance in obese subjects compared with controls [62], while other evidence suggests a lack of significant between-group differences [63] or better performance in obese patients [64].

Experimental studies involving subjects with Class III obesity (formally known as “morbid obesity”) are limited [34,65,66]. In this population, a number of comorbidities may independently affect cognitive performance. Indeed, obesity increases, per se, the risk of cardio-cerebrovascular diseases, diabetes, and metabolic syndrome; this could sully the relationship between obesity and cognition [67,68]. However, it has been documented that cognitive inefficiency can also occur independently of medical comorbidities [48]. In addition, one should also note that many studies have failed to mention relevant data characterizing the study sample (e.g., sex, age, education, and general clinical status). Therefore, it is unclear whether obesity predisposes to cognitive failures, especially considering the possible confounding/mediating effects of covariates.

To disentangle the link binding obesity to EFs, the current study aimed at verifying whether differences exist on general executive functioning and selective executive subdomains (e.g., inhibitory control, verbal fluency, and psychomotor speed) between treatment-seeking overweight, obese, and morbidly obese patients, using normal-weight subjects as control participants. Particularly, we checked the modulating effects of sociodemographic characteristics (i.e., sex, age, and years of formal education) and morbidity indexes. Since obesity is a clinical condition that is often accompanied by a number of chronic diseases likely inducing neurofunctional changes, we expect to find a relationship between obesity and executive performance. In particular, we hypothesize that BMI-ranked groups, i.e., from normal-weight to morbid obesity, show different performances in the executive tasks employed.

## 2. Methods

### 2.1. Participants

Three hundred and four subjects agreed to participate in this study. Eighty were morbidly obese patients (sixty females, M age = 36.20, SD = 13.85; M education = 9.65, SD = 3.67; M BMI = 46.18, SD = 5.11), seventy-six were obese patients (thirty-six females, M age = 38.00, SD = 11.82; M education = 10.58, SD = 2.98; M BMI = 34.17, SD = 2.20), fifty-two were overweight patients (twenty females, M age = 34.38, SD = 10.57; M education = 13.00, SD = 0.86; M BMI = 27.25, SD = 1.10), and ninety-six were normal-weight subjects that served as controls (fifty-two females, M age = 30.00, SD = 6.76; M education = 12.58, SD = 1.39; M BMI = 23.74, SD = 1.55). Patients were recruited at the Department of Experimental Medicine (outpatient clinic of dietetics, sports medicine, and psychophysical health) of the University of Campania “Luigi Vanvitelli” (Italy). Inclusion criteria were age ≥18 years and formal education ≥5 years. Furthermore, all patients (from overweight to morbidly obese individuals) underwent at least the first outpatient examination, and they had been prescribed a low-calorie diet aimed at weight loss. Exclusion criteria were: BMI < 18.5 (i.e., underweight), previous or current history of intellectual and/or linguistic deficits, previous or current history of psychopathological, psychiatric or neurocognitive disorders (including non-progressive or metabolic dementia), history of alcohol or substance abuse/addiction, or ongoing pharmacological treatment with drugs interfering with cognitive abilities. 

### 2.2. Materials and Procedure

For each participant, we collected demographic information, i.e., sex, age and education (years of education were recorded in accordance with the Italian schooling system: 5 years = primary school; 8 years = secondary school; 13 years = high school; 16 years = bachelor’s degree; 18 years = master’s degree; 20 years = graduate school, PhD), and anthropometric measures (i.e., BMI). Therefore, participants were administered a neuropsychological battery including a screening test of general executive functioning (i.e., the Frontal Assessment Battery–15), and three executive tasks assessing impulsivity/inhibitory control (i.e., Stroop Color-Word Test), cognitive flexibility (i.e., FAS verbal fluency test), and psychomotor speed (i.e., Digit Symbol Substitution Test), respectively. Finally, the Cumulative Illness Rating Scale was used for computing the number of concurrent diseases and related severity. 

*Frontal Assessment Battery–15* (FAB15) [69]. The FAB15 is a short cognitive screening battery exploring general executive functioning (abstraction, generativity, cognitive flexibility, planning, sensitivity to interference, and inhibitory control). The scoring range is 0–15 and a higher score reflects better performance. 

*Stroop Color-Word Test* (SCWT) [70]. This task assesses the ability to inhibit interference from a dominant response tendency. Moreover, it appears to tap other cognitive domains such as attention, processing speed, cognitive flexibility, and working memory [71,72]. The interference effect on both execution time (Stroop-T) and errors (Stroop-E) served as dependent variables. 

*FAS Verbal Fluency Test* (FAS) [59,73,74]. It is a test assessing phonemic verbal fluency, which requires participants to produce as many words as possible that begin with letters “F”, “A” and “S” (omitting personal proper nouns, surnames, and place names). The total number of produced words represents the dependent variable. The FAS fluency is considered a valid measure of selective attention, set-shifting, generativity, and self-monitoring abilities.

*Digit Symbol Substitution Test* (DSST) [75,76]. The DSST is a pencil-and-paper test for assessing psychomotor speed; however, it is widely used to explore alternative domains such as processing speed, set-shifting, working memory, and associative and implicit learning. More specifically, participants are presented with a grid of numbers and matching symbols under which there is a test section with numbers and empty boxes. The task consists of filling as many empty boxes as possible with the appropriate symbol. The number of correct number-symbol matches completed in 90 sec is scored and entered as dependent variable. 

*Cumulative Illness Rating Scale* (CIRS) [77]. The CIRS consists of fourteen categories related to different body systems and scores the severity of each condition on a five-point (0–4) Likert scale. Comorbidity and severity indexes entered as dependent variables. 

### 2.3. Statistical Analyses

The assumptions of the Generalized Linear Model (GLM) were verified by checking univariate and multivariate normality. For descriptive purposes, we ran parametric (univariate Analysis of Variance, ANOVA) and non-parametric analyses (two-way chi-squared test, χ^2^), when needed. In addition, correlation analysis (Spearman’s correlation, ρ_s_) was used to quantify the relationships between the variables under examination. The effects of sex, age, education, and morbidity (e.g., number of illnesses and the respective severity) were taken into account. Indeed, multivariate Analysis of Covariance (MANCOVA) was performed, with the five executive scores entering the model as dependent variables, the BMI subgroups (normal-weight, overweight, obese, and morbidly obese) as fixed factors, and sex, age, education, CIRS-morbidity, and CIRS-severity scores as covariates. Any post-hoc analysis was performed according to Bonferroni’s correction for multiple comparisons (*p*_bonf_). Eta squared (*η*^2^) or partial eta squared ($\eta_{p}^{2}$) were used to quantify the effect sizes. Statistical analyses were conducted by using IBM SPSS v. 26 and JASP packages. 

## 3. Results

To assess univariate normality, skewness and kurtosis indexes were assessed. Values ranging from −2 and +2 suggest the absence of appreciable deviations from normality. Square root transformation (√X_i_) was applied to normalize variables in line with skewness index (i.e., |1|< γ <|2|). Univariate outliers, i.e., z-scores higher than 3 in absolute terms, were removed. For multivariate diagnostics of outliers, Mahalanobis’ distance ($D_{i}^{2}$) was calculated. Accordingly, no multivariate outliers were detected (mean $D_{i}^{2}$ = 9.92, df = 10, *p*_s_ > 0.001). Multivariate normality was assumed by Mardia’s coefficient $\left(\frac{\sum_{i=1}^{N}{\left(D_{i}^{2}\right)}^{2}\;}{N}\right)$ = 0.33 < 120. Analysis of missing data revealed random missingness (MCAR) that was handled via the recommended multiple imputation method [78,79].

### 3.1. Descriptive Statistics

Sample characteristics are summarized in Table 1. A difference was found in the frequency of gender levels between the four BMI subgroups (χ^2^_(3)_ = 20.50, *p* < 0.001, φ = 0.26). Particularly, according to the analysis of adjusted-standardized residuals (z_r_) [80], the number of females was significantly lower than expected (z_r_ = −2.7) in the overweight subgroup; conversely, the number of females was significantly higher than expected (z_r_ = 4.1) within the morbidly obese patients. Results of univariate ANOVA showed that female participants had a higher BMI than males [F_(1, 302)_ = 11.90, *p* = 0.01, η^2^ = 0.06; female, M BMI = 34.51, SD = 10.22 vs. male, M BMI = 30.77, SD = 7.79]. No gender difference was found on the FAB15 score [F_(1, 302)_ = 0.14, *p* = 0.20], DSST score [F_(1, 302)_ = 3.21, *p* = 0.07], and Stroop-E [F_(1, 302)_ = 0.41, *p* = 0.52], while differences emerged on the FAS score [F_(1, 302)_ = 5.48, *p* = 0.02, η^2^ = 0.03] and Stroop-T [F_(1, 302)_ = 7.66, *p* = 0.06, η^2^ = 0.02]. As for the FAS score, females outperformed males, whereas the opposite pattern was observed for the Stroop-T score. 

A significant age effect was found among BMI subgroups [F_(3, 300)_ = 8.70, *p* < 0.001, η^2^ = 0.08]. Post-hoc analysis revealed that obese (mean diff. = 8.00, SE = 1.67, *p*_bonf_ < 0.001) and morbidly obese patients (mean diff. = 6.20, SE = 1.65, *p*_bonf_ = 0.01) were older than normal-weight controls. As for the education variable, a between-group difference emerged [F_(3, 300)_ = 29.41, *p* < 0.001, η^2^ = 0.23]; more specifically, normal-weight (vs. obese, mean diff. = 2.00, SE = 0.39, *p*_bonf_ < 0.001; vs. morbidly obese, mean diff. = 2.93, SE = 0.38, *p*_bonf_ = < 0.001) and overweight (vs. obese, mean diff. = 2.42, SE = 0.45, *p*_bonf_ < 0.001; vs. morbidly obese, mean diff. = 3.35, SE = 0.45, *p*_bonf_ < 0.001) subjects were more educated than obese and morbidly obese patients. 

Finally, about the CIRS scores, we found a statistically significant difference on both CIRS-morbidity [F_(3, 300)_ = 235.25, *p* < 0.001, η^2^ = 0.56] and CIRS-severity scores [F_(3, 300)_ = 346.47, *p* < 0.001, η^2^ = 0.69], with a progressive increase of multimorbidity and severity from normal-weight to morbid obesity (all *p*_bonf_ < 0.001). 

### 3.2. Gender Differences in Each BMI Subgroup

In the normal-weight group, female participants were older [F_(1, 95)_ = 10.99, *p* = 0.001, η^2^ = 0.10; female, M age = 32.00 years, SD = 7.68 vs. male, M age = 27.64 years, SD = 4.51] and more educated [F_(1, 95)_ = 11.31, *p* = 0.001, η^2^ = 0.11; female, M education = 13.00 years, SD = 0.05 vs. male, M education = 12.09 years, SD = 1.95] than male participants. As for cognitive scores and morbidity indexes, no gender differences were detected (all *p*_s_ > 0.09). 

In the overweight group, females were younger as compared with males [F_(1, 50)_ = 6.16, *p* = 0.02, η^2^ = 0.11; female, M age = 30.00 years, SD = 6.71 vs. male, M age = 37.12 years, SD = 1.67]. Instead, no difference was found on the education variable (*p* = 0.99). As concerns performance on executive tasks, males got higher scores than females on the FAB15 [F_(1, 50)_ = 21.41, *p* < 0.001, η^2^ = 0.36; female, M = 12.50, SD = 1.71 vs. male, M = 14.17, SD = 0.38]; conversely, females performed better than males on both Stroop-E [F_(1, 50)_ = 6.43, *p* = 0.01, η^2^ = 0.11; female, M = 0.20, SD = 0.25 vs. male, M = 0.94, SD = 1.28] and Stroop-T [F_(1, 50)_ = 5.78, *p* = 0.02, η^2^ = 0.10; female, M = 9.45, SD = 5.40 vs. male, M = 13.87, SD = 7.03]. We did not find sex differences on FAS and DSST scores, not even on morbidity indexes (all *p*_s_ > 0.06). 

In the obese group, female participants were older [F_(1, 74)_ = 8.10, *p* = 0.006, η^2^ = 0.10; female, M age = 41.89 years, SD = 10.10 vs. male, M age = 34.50 years, SD = 12.27] and more multimorbid than males [F_(1, 74)_ = 10.57, *p* = 0.002, η^2^ = 0.20; female, M = 2.35, SD = 0.71 vs. male, M age = 1.71, SD = 0.56]. No additional differences were observed on the other variables of interest (all *p*_s_ > 0.09). 

In the morbidly obese group, females were older than males [F_(1, 78)_ = 9.36, *p* = 0.003, η^2^ = 0.11; female, M age = 38.80 years, SD = 13.20 vs. male, M age = 28.40 years, SD = 13.08] but no sex differences emerged with regard to formal schooling (*p* = 0.83). In terms of executive functioning, gender differences were found on verbal fluency and inhibition domains, with females getting higher scores on the FAS [F_(1, 78)_ = 52.66, *p* < 0.001, η^2^ = 0.74; female, M = 35.33, SD = 0.49 vs. male, M = 27.50, SD = 3.74] but performed worse than males on both Stroop-E [F_(1, 78)_ = 4.89, *p* = 0.03, η^2^ = 0.06; female, M = 0.82, SD = 1.00 vs. male, M = 0.25, SD = 0.45] and Stroop-T [F_(1, 78)_ = 14.42, *p* < 0.001, η^2^ = 0.16; female, M = 22.63, SD = 12.59 vs. male, M = 11.20, SD = 8.13]. ANOVA did not reveal sex differences on FAB15 and DSST scores, not even on morbidity indexes (all *p*_s_ > 0.15). 

### 3.3. Correlation Analysis

The Spearman’s correlation coefficients are tabulated and reported in Table 2. BMI strongly correlated with CIRS-morbidity (ρ_s_ = 0.892, *p* < 0.001) and CIRS-severity (ρ_s_ = 0.888, *p* < 0.001); it also showed a negative moderate correlation with year of education (ρ_s_ = −0.468, *p* < 0.001). Still, BMI showed weak and negative correlations with DSST (ρ_s_ = −0.293, *p* < 0.001), FAS (ρ_s_ = −0.273, *p* < 0.001) and FAB15 (ρ_s_ = −0.173, *p* < 0.05); conversely, positive correlations were found with Stroop-E (ρ_s_ = 0.188, *p* < 0.01), Stroop-T (ρ_s_ = 0.180, *p* < 0.01) and age (ρ_s_ = 0.136, *p* < 0.05). Age correlated moderately, in negative direction, with DSST (ρ_s_ = −0.532, *p* < 0.001) but positively with Stroop-T (ρ_s_ = 0.575, *p* < 0.001) and Stroop-E (ρ_s_ = 0.411, *p* < 0.001). In addition, weak positive correlations were found with CIRS-severity (ρ_s_ = 0.251, *p* < 0.001) and CIRS-morbidity (ρ_s_ = 0.231, *p* < 0.001). Finally, moderate negative correlations were shown between years of education and CIRS (CIRS-severity, ρ_s_ = −0.492, *p* < 0.001; CIRS-morbidity, ρ_s_ = −0.451, *p* < 0.001); moreover, education correlated positively with FAB15 (ρ_s_ = 0.531, *p* < 0.001), DSST (ρ_s_ = 0.456, *p* < 0.001) and FAS (ρ_s_ = 0.449, *p* < 0.001). A negative moderate correlation was found with Stroop-T (ρ_s_ = −0.362, *p* < 0.001) and Stroop-E (ρ_s_ = −0.362, *p* < 0.001).

### 3.4. Analysis of Variance for Executive Scores

Between-group differences were found on FAB15 [F_(3, 300)_ = 27.78, *p* < 0.001, η^2^ = 0.30], FAS [F_(3, 300)_ = 21.61, *p* < 0.001, η^2^ = 0.24], DSST [F_(3, 300)_ = 26.76, *p* < 0.001, η^2^ = 0.21], and Stroop-T scores [F_(3, 300)_ = 10.93, *p* < 0.001, η^2^ = 0.10]; no difference was detected on the Stroop-E [F_(3, 300)_ = 0.85, *p* = 0.47] (see Table 1). Multiple comparisons post-hoc analysis revealed that both obese and morbidly obese patients reported lower performance on FAB15 than normal-weight and overweight subjects (all *p*_bonf_ < 0.001); moreover, patients with morbid obesity got lower FAB15 scores than obese patients (mean diff. = −1.91, SE = 0.47, *p*_bonf_ < 0.001). As concerns FAS, DSST, and Stroop-T scores, both obese and morbidly obese patients obtained lower scores than normal-weight and overweight participants (all *p*_bonf_ < 0.001); however, no differences were detected between the two obese subgroups (FAS, mean diff. = |2.60|, SE = 2.49; DSST, mean diff. = |2.60|, SE = 2.40; Stroop-T, mean diff. = |1.74|, SE = 2.49). 

### 3.5. Multivariate Analysis of Covariance 

Results of MANCOVA (Table 3) showed a significant effect of BMI [F_(15, 348)_ = 5.54, *p* < 0.001, $\eta_{p}^{2}$ = 0.19] even after adjusting for the significant contribution of sociodemographic variables [sex, F_(5, 114)_ = 3.80, *p* = 0.003, $\eta_{p}^{2}$ = 0.14; age, F_(5, 114)_ = 20.91, *p* < 0.001, $\eta_{p}^{2}$ = 0.48; education, F_(5, 114)_ = 13.45, *p* < 0.001, $\eta_{p}^{2}$ = 0.37] and CIRS-severity score [F_(5, 114)_ = 2.64, *p* = 0.03, $\eta_{p}^{2}$ = 0.10]; the CIRS-morbidity score was not a significant covariate [F_(5, 114)_ = 1.35, *p* = 0.24]. More specifically, between-group differences were found on the FAB15 [F_(3, 160)_ = 20.58, *p* < 0.001, $\eta_{p}^{2}$ = 0.28], FAS [F_(3, 160)_ = 11.51, *p* < 0.001, $\eta_{p}^{2}$ = 0.18], DSST [F_(3, 160)_ = 6.18, *p* = 0.001, $\eta_{p}^{2}$ = 0.10], and Stroop-T [F_(3, 160)_ = 5.66, *p* = 0.001, $\eta_{p}^{2}$ = 0.10]; conversely, no difference between BMI subgroups on the Stroop-E [F_(3, 160)_ = 0.86, *p* = 0.46] were shown. 

Pairwise comparisons based on Bonferroni’s correction showed no significant difference between obese patients and normal- and over- weight subjects in any of the executive scores (p_bonf_ range = 0.08–0.99). As concerns patients with morbid obesity, they got lower FAB15 scores than normal-weight (mean diff. = −3.75, SE = 0.52, *p*_bonf_ < 0.001), overweight (mean diff. = −4.99, SE = 0.54, *p*_bonf_ < 0.001), and obese participants (mean diff. = −3.00, SE = 0.53, *p*_bonf_ < 0.001). Still, morbidly obese patients obtained lower FAS scores as compared with normal-weight (mean diff. = −10.94, SE = 2.87, *p*_bonf_ = 0.001) and overweight subjects (mean diff. = −9.60, SE = 3.01, *p*_bonf_ = 0.01); however, no difference was found between obese and morbidly obese patients on this test (mean diff. = −2.33, SE = 2.95, *p*_bonf_ = 0.25). Similarly, patients with morbid obesity reported lower DSST and higher Stroop-T scores as compared with normal-weight (DSST, mean diff. = −11.38, SE = 3.54, *p*_bonf_ < 0.001; Stroop-T, mean diff. = 8.62, SE = 2.19, *p*_bonf_ = 0.001) and overweight subjects (DSST, mean diff. = −10.99, SE = 3.75, *p*_bonf_ = 0.001; Stroop-T, mean diff. = 7.09, SE = 2.29, *p*_bonf_ = 0.01) while no difference was detected if compared with obese participants (DSST, mean diff. = 1.92, SE = 4.47, *p*_bonf_ = 0.14; Stroop-T, mean diff. = 5.77, SE = 2.24, *p*_bonf_ = 0.09). 

## 4. Discussion

The current study was designed to compare the performance of morbidly obese, obese, overweight, and normal-weight participants on neuropsychological tasks exploring general executive functioning and specific (i.e., inhibitory control, verbal fluency, and psychomotor speed) executive subdomains. Our study encompassed the simultaneous contribution of sociodemographic variables (sex, age, and formal schooling) and comorbidity/severity indexes as covariates in a GLM. 

In a scientific context in which the link between EFs and obesity needs to be further investigated [7,25,51,52,53,54,81], our findings showed that morbidly obese individuals got poor executive scores regardless of the significant effects of the above-mentioned covariates. Notably, in line with previous studies [50], we found no significant differences between obese, overweight, and normal-weight subjects on the executive domains explored. This evidence suggests that severe obesity, more than obesity, may represent a clinical condition that impacts on cognition. Although a few contributions are available on this topic [34,65,66], their results converge on the idea that morbidly obese patients, net of the presence of eating disorders, would show weaknesses in tasks assessing motor planning, verbal fluency, processing speed, attention/vigilance, and inhibition. 

Our results showed that morbidly obese patients got lower FAB15 scores (i.e., lower general executive functioning) than normal-weight, overweight, and obese participants. Therefore, the FAB15 may be sensitive enough to detect executive blunting in morbid obesity and to differentiate morbidly obese from obese patients in terms of executive functioning. Morbidly obese patients scored worse on FAS, DSST and Stroop-T as compared with normal-weight and overweight subjects, although they got similar performance than obese patients.

As concerns the executive subdomains explored, poor performance on the FAS test underlies low self-monitoring/set-shifting capabilities. Reduced psychomotor speed (i.e., DSST) highlights some difficulties in detecting and responding appropriately to rapid changes in the environment. Finally, longer latency time in the Stroop Test suggests inadequate inhibitory control and thus decreased ability to voluntarily suppress interfering information or prepotent habitual responses.

From an anatomofunctional standpoint, the integrity of these domains seems to depend on a large frontal network including (i) anterior and posterior cingulate cortices, inferior frontal gyrus and medial frontal areas for verbal fluency, (ii) dorsolateral, ventromedial, orbital prefrontal and anterior cingulate cortices for inhibitory control, and (iii) the frontoparietal network, especially the middle frontal gyrus and the posterior parietal cortex, for psychomotor speed. Great interest is held by the functional connectivity within the PFC, as well as between PFC and subcortical structures (e.g., basal ganglia, subthalamic nucleus, hippocampal formation) in patients with and without eating disorders [82,83,84,85,86,87,88]. The projections from the PFC towards hypothalamic circuits appear to be involved in the modulation of hunger and satiety signals, whereas the striatal and ventral midbrain circuits are relevant for reward processing [83,89,90]. In morbidly obese patients, likely also due to the more complicated clinical profile (e.g., metabolic syndrome) [91,92,93], abnormal activity within these neural circuits might affect EFs and lead to difficulties in planning regular eating patterns, inability to delay gratification, or inhibit prepotent responses to highly palatable foods [53]. 

To better understand the relationship between obesity and cognition, there is a need to examine the interaction between obesity and brain physiology. Many studies have found that obesity in middle age correlated with an increased risk of cognitive decline in later life [94]. Indeed, excessive body fat seems to be associated with reduced brain volume in cognitively healthy older adults, but in patients with cognitive deficits, it exerts an additional detrimental effect [95]. More specifically, greater body adiposity and elevated waist-hip ratio were found to be related with generalized structural alterations involving orbital frontal cortex, temporal and parietal cortices, and hippocampus [96,97,98]. In a structural brain mapping cohort study of patients with Mild Cognitive Impairment and Alzheimer’s disease (AD), a higher BMI value was found to be correlated with brain volume reduction in the frontal, temporal, and parietal cortices (0.5–1.5%) per one unit increase in BMI [44]. In line with this vein, longitudinal studies have highlighted a significant increase in temporal lobe atrophy between 13 and 16% for each one-unit increment in BMI [45]. Still, brain scanning evidence has suggested that obese individuals showed lower gray matter density in the middle frontal gyrus, primary somatosensory cortex, and putamen than normal-weight controls [99,100].

Although the mechanisms underlying the relationship between obesity and cognitive functions need to be further investigated, physiology research has identified a reliable mechanism of action in the activity of the neuro-immune-endocrine systems. Adipose tissue fulfills neuro-immune-endocrine functions, participating in the homeostasis [101]. In obesity, adipose tissue produces cytokines such as interleukin (specifically IL1ß and IL6), interferon γ (IFNγ), Tumor Necrosis Factor α (TNFα), and Monocyte Chemoattractant Protein 1 (MCP1), which promote chronic inflammation [102,103,104,105,106,107]. Hypertrophy of adipocytes induces a state of chronic inflammation that causes changes in the brain by inducing neuroinflammation. For instance, some studies have shown that inflammatory processes, especially in the hypothalamus, often follow high-fat feeding [15,108,109]. Moreover, chronic low-grade inflammation alters the blood-brain barrier (BBB) due to endothelial dysfunction, generating neuroinflammation and increasing oxidative stress, often resulting in cognitive impairment [110,111]. Since the BBB interfaces the periphery with the central nervous system, it could represent the bridge between peripheral inflammation and obesity-related differences in cognitive processes. Studies on obese animal models and human obese patients have shown a relationship between diet and cognitive functioning, especially in working memory and learning [110,111,112].

Furthermore, systemic inflammation could predict cognitive decline and dementia [113,114,115,116]. Adipose tissue may also affect β-amyloid metabolism [117,118]. Indeed, some neuropathological features of AD (e.g., amyloid plaques, neurofibrillary tangles) appear to be most observed in obese compared with normal-weight older adults [119]. In addition, a higher concentration of amyloid plaques has been detected in the hippocampal formation of cognitive intact morbidly obese patients when compared with non-obese subjects [120,121]. A high-fat diet may increase either body weight or cognitive impairment [122]. Longitudinal data have shown that higher caloric intake was related to an increased risk of AD at 6.3 years follow-up [123]. In addition, high-fatty acids diets, or diets rich in simple sugars, may undermine brain physiology, harming the integrity of the blood–brain barrier (BBB) [124], protecting the central nervous system from bloodborne toxins. AD and vascular dementia are linked to BBB dysfunction [125] and longitudinal studies sustained a significant relationship between midlife obesity and lower BBB functioning [45].

This study supports the hypothesis that patients with morbid obesity show inefficiencies in executive functioning, including when the contributions of sociodemographic factors and comorbidity indexes are taken into account. The main limitation of the present study is that we used BMI as the sole indicator of obesity, although previous research has shown that it is not a good indicator of obesity, besides that its relationship to EFs is notoriously mixed. BMI is unable to discriminate between muscle and adipose tissues and cannot assess regional adiposity [126]. Conversely, waist circumference or waist-to-hip ratio (WHR) could be superior to BMI as measures of obesity since they showed higher correlations with obesity-related risk factors [127,128,129]. Furthermore, robust evidence is available on the predictive role of WHR for executive performance in obese patients [49]. An additional limitation, in terms of external validity, is that we tested treatment-seeking overweight, obese, and morbidly obese patients; therefore, our results are not generalizable to patients not requiring a dietary intervention. Finally, one should consider that, although some cognitive tasks assessing EFs are based on a verbal response set (e.g., verbal fluency, Stroop test), most executive tests require hand motor responses to be properly performed (e.g., the DSST) [130,131]. Thus, it could be interesting to test the mediation effects exerted by hand motor function in the relationship between EFs and obesity. 

## 5. Conclusions

Our results support the relationship between severe/morbid obesity and poor EFs, also considering the contribution of sociodemographic variables and the severity of concurrent diseases. Specifically, results from the present study suggest that: (1) patients with morbid obesity demonstrate decreased general executive functioning compared with normal-weight, overweight, and obese participants; (2) morbidly obese patients score worse than normal-weight and overweight subjects on cognitive tasks exploring selective executive subdomains, namely, cognitive flexibility, psychomotor speed, and inhibition processes; (3) EFs need to be accounted into the management of obese patients due to the non-negligible clinical relevance in diagnostic, therapeutic, and prognostic terms; (4) the direction of the relationship between obesity and executive defects needs to be further investigated. The study of this matter remains a strong challenge for future research. 

## Figures and Tables

**Table 1:** Sample characteristics.

Characteristics	Normal-Weight (*n* = 96)	Overweight (*n* = 52)	Obese (*n* = 76)	Morbidly Obese (*n* = 80)	*p*-Value
BMI, mean (SD)	23.74 (1.55)	27.25 (1.10)	34.17 (2.20)	46.18 (5.11)	
Sex (f/m)	52/44	20/32	36/40	60/20	
Age in years, mean (SD)	30.00 (6.76)	34.38 (10.57)	38.00 (11.82)	36.20 (13.85)	<0.001
Education in years, mean (SD)	12.58 (1.39)	13.00 (0.86)	10.58 (2.98)	9.65 (3.67)	<0.001
CIRS-morbidity, mean (SD)	0.23 (0.43)	1.11 (0.32)	2.04 (0.71)	3.43 (0.62)	<0.001
CIRS-severity, mean (SD)	0.05 (0.10)	0.26 (0.07)	1.38 (0.13)	1.67 (0.43)	<0.001
FAB15, mean (SD)	13.00 (1.76)	13.50 (1.38)	11.41 (2.68)	9.50 (0.59)	<0.001
FAS, mean (SD)	45.35 (6.93)	44.75 (9.78)	34.80 (13.23)	32.20 (4.56)	<0.001
DSST, mean (SD)	59.04 (12.03)	61.15 (1.42)	45.55 (20.57)	43.50 (13.82)	<0.001
Stroop-T, mean (SD)	13.43 (8.17)	12.17 (6.75)	18.04 (8.05)	19.77 (12.61)	<0.001
Stroop-E, mean (SD)	0.52 (1.12)	0.65 (1.07)	0.78 (1.08)	0.69 (0.94)	0.47

CIRS: Cumulative Illness Rating Scale; BMI: Body Mass Index; FAB15: Frontal Assessment Battery–15; FAS: FAS Verbal Fluency Test; DSST: Digit Symbol Substitution Test; Stroop-T: Stroop Color-Word Test-Time; Stroop-E: Stroop Color-Word Test-Error.

**Table 2:** Correlation Matrix.

	BMI	Age	Education	CIRS-Morbidity	CIRS-Severity	FAB15	FAS	DSST	Stroop-E	Stroop-T
BMI	–									
Age	0.136 *	–								
Education	−0.468 ***	−0.085	–							
CIRS-morbidity	0.892 ***	0.231 ***	−0.451 ***	–						
CIRS-severity	0.888 ***	0.251 ***	−0.492 ***	0.944 ***	–					
FAB15	−0.173 *	−0.106	0.531 ***	−0.128	−0.165 *	–				
FAS	−0.273 ***	−0.030	0.449 ***	−0.202 *	−0.235 **	0.563 ***	–			
DSST	−0.293 ***	−0.532 ***	0.456 ***	−0.269 ***	−0.280 ***	0.490 ***	0.378 ***	–		
Stroop-E	0.188 **	0.411 ***	−0.304 ***	0.260 ***	0.220 **	−0.520 ***	−0.258 ***	−0.432 ***	–	
Stroop-T	0.180 **	0.575 ***	−0.362 ***	0.304 ***	0.299 ***	−0.267 ***	−0.268 ***	−0.659 ***	0.456 ***	–

BMI: Body Mass Index; FAB15: Frontal Assessment Battery–15; FAS: FAS Verbal Fluency Test; DSST: Digit Symbol Substitution Test; Stroop-E: Stroop Color-Word Test-Error; Stroop-T: Stroop Color-Word Test-Time. Note: * *p* <0.05; ** *p*<0.01; *** *p* < 0.001.

**Table 3:** Analyses of Covariance.

	Sum of Square	*F*	*p*-Value
BMI × FAB15	167.51	20.58	<0.001
Sex	7.62	4.07	<0.05
Age	2.87	1.53	0.06
Education	72.58	38.76	<0.001
CIRS-morbidity	8.63	4.61	<0.05
CIRS-severity	7.54	4.02	<0.05
BMI × FAS	2881.93	11.51	<0.001
Sex	2.68	0.05	0.77
Age	36.16	0.71	0.95
Education	72.58	21.04	<0.001
CIRS-morbidity	25.83	0.51	0.48
CIRS-severity	48.97	0.96	0.33
BMI × DSST	3558.23	6.18	<0.01
Sex	9.94	0.12	0.73
Age	4554.88	55.73	<0.001
Education	3123.28	38.21	<0.001
CIRS-morbidity	81.52	1.00	0.32
CIRS-severity	115.70	1.42	0.24
BMI × Stroop-T	820.61	5.66	<0.01
Sex	164.17	5.92	<0.05
Age	1599.91	57.72	<0.001
Education	24.07	0.87	0.35
CIRS-morbidity	2.95	0.11	0.74
CIRS-severity	133.59	4.82	<0.05
BMI × Stroop-E	2.77	0.86	0.46
Sex	1.57	4.12	<0.05
Age	22.10	58.15	<0.001
Education	8.82	23.20	<0.001
CIRS-morbidity	1.74	4.57	<0.05
CIRS-severity	4.13	10.88	<0.01

CIRS: Cumulative Illness Rating Scale; BMI: Body Mass Index; FAB15: Frontal Assessment Battery–15; FAS: FAS Verbal Fluency Test; DSST: Digit Symbol Substitution Test; Stroop-T: Stroop Color-Word Test-Time; Stroop-E: Stroop Color-Word Test-Error.

## Data Availability

The data presented in this study are available on request from the corresponding author.
